# Uptake of Recommended Vaccines and Its Associated Factors Among Malaysian Pilgrims During Hajj and Umrah 2018

**DOI:** 10.3389/fpubh.2019.00268

**Published:** 2019-09-18

**Authors:** Mohammed Dauda Goni, Nyi Nyi Naing, Habsah Hasan, Nadiah Wan-Arfah, Zakuan Zainy Deris, Wan Nor Arifin, Aisha Abubakar Baaba

**Affiliations:** ^1^Department of Microbiology and Parasitology, School of Medical Sciences, Universiti Sains Malaysia, Kubang Kerian, Malaysia; ^2^Faculty of Medicine, Universiti Sultan Zainal Abidin, Medical Campus, Kuala Terengganu, Malaysia; ^3^Faculty of Health Sciences, Universiti Sultan Zainal Abidin, Gong Badak Campus, Kuala Terengganu, Malaysia; ^4^Unit of Biostatistics and Research Methodology, School of Medical Sciences, Universiti Sains Malaysia Health Campus, Kubang Kerian, Malaysia; ^5^Centre for Language Studies and Generic Development, Universiti Malaysia Kelantan, Kota Bharu, Malaysia

**Keywords:** influenza vaccine, pneumococcal vaccine, pilgrims, Hajj, Umrah

## Abstract

This study aimed to assess the uptake of recommended vaccines and to identify the factors associated with the vaccines' uptake among Malaysian Hajj and Umrah pilgrims. A cross-sectional survey among Malaysian Hajj and Umrah pilgrims in 2018. The uptake of the recommended vaccines was surveyed through an anonymous self-administered questionnaire to pilgrims attending a pre-departure Hajj/Umrah orientation course. Descriptive statistics were used for elaborating the demographic characteristics and vaccines uptake of the respondents. Multiple logistic regression was used for predicting the factors associated with the vaccines' uptake. A total of 1,274 pilgrims participated in the study with a mean age (standard deviation) of 42.42 (15.6). A total of 833 (65.4%) participants were females and 232 of the participants (18.2%) had at least more than one chronic disease. The uptake of influenza and pneumococcal vaccines were 28.6% (364/1,274) and 25.4% (324/1,274), respectively. Among the 527 pilgrims who were “at increased risk” of infections, 168 (31.9%) and 184 (34.9%) received influenza and pneumococcal vaccines, respectively. Gender, marital status and occupation were the common predictors associated with vaccines uptake. The vaccination uptake among Malaysian Hajj and Umrah pilgrims is low and declining from previous years. Educating the pilgrims toward vaccine uptake is essential and exploring the barriers for vaccination.

## Introduction

The Muslims Holy pilgrimage to Makkah and Madinah, Saudi Arabia is an annual event that records about 3 million pilgrims for Hajj and nearly 5 million pilgrims participating for the Minor Hajj known as Umrah conducted through the year (1, 2). Hajj pilgrims from more than 180 countries across the globe visit Saudi Arabia for the 2 pilgrimages yearly (3). These mass gatherings are characterized by many challenges like an increased risk of infection particularly the respiratory tract infections due to the enormous population density of the participants, shared accommodation, environmental pollution and extreme climatic conditions (4, 5). Outbreaks of diseases like influenza and cholera have all been associated with these two events (6). Recently, Hajj pilgrimage has also drawn considerable attention from participating countries on the possible spread and transmission of emerging infections such as the Middle East respiratory syndrome coronavirus (MERS-CoV) due to numerous cases recorded by the host country since its emergence in 2012 and pandemic H1N1 (7).

Two studies conducted in 2009 and 2013 reported a high prevalence of respiratory illness symptoms among Malaysian pilgrims at 90 and 93.4%, respectively and the majority of the pilgrims reported contracting infections at Arafat (8). The proportion of respiratory tract infections was lower (38.9%) in those with influenza vaccine uptake when compared to unvaccinated pilgrims (61.1%) (9).

The pre-travel vaccination is a major essential component that reduces the risk of vaccine-preventable diseases as recommended by the World Health Organization (WHO) (10). Similarly, it is vital in preventing the risk of transmission of the infections from pilgrims returning to their home countries with the bacteria carriage (11). Several studies have demonstrated the effectiveness of the recommended vaccines. Egyptian pilgrims recorded low prevalence for influenza A (0%) following strict compliance with pre-Hajj influenza vaccination (12). An enhanced surveillance of Hajj pilgrims from Australia reported a prevalence rate of 1.1% from unvaccinated pilgrims (13). Vaccination coupled with other prevention practices during Hajj and Umrah such as the use of face mask, hand hygiene and cough etiquette are essential in ensuring a healthy pilgrimage (14). Saudi government usually liaises with health authorities from participating countries on the vaccine recommendation (15).

In Malaysia, meningococcal quadrivalent (ACYW-135) polysaccharide vaccine is compulsory for all intending Hajj pilgrims in line with the Saudi Arabia Government requirement. The vaccines are to be administered at least 2 weeks before departure to the Holy Land. However, this vaccine confers immunity to an individual for 2 years (16, 17). Malaysian Government bears the responsibility of providing the meningococcal vaccine free of charge to the pilgrims. In addition, the Malaysian Health authorities recommend intending Hajj pilgrims to take influenza and pneumococcal vaccines to augment their body immunity system. Both vaccines are strongly encouraged for pilgrims aged 50 years and above and those with underlying medical conditions like asthma, diabetics, lung or kidney diseases. The vaccine should be administered at least a month before the departure date to the Holy Land and can confer immunity for influenza and pneumococcal infections for 1 and 5 years, respectively (18).

However, many studies have found varying figures in the uptake of the recommended vaccines ranging from 0.7% up to 100% among Hajj and Umrah pilgrims despite the recommendations by health authorities (3, 19–23). Full vaccination coverage was recorded during the 2009 influenza pandemic following Saudi authorities' insistence on vaccination (24). This is mainly due to the misconception by many people that vaccines themselves could revert to cause disease, reliance on natural immunity and insufficient awareness (25, 26). However, the common belief for alternative medicine during Hajj through consumption dates, honey, olive, pomegranates, and other blessed foods and fruits in Islam are common resulting in vaccine hesitancy. This misconception and misperception are generated and spread in Malaysia and affect the uptake of the recommended vaccines (27). The main purpose of this study is to evaluate the uptake of the recommended vaccine among Malaysian Hajj pilgrims in the year 2018 and to determine the association between socio-demographic variables, comorbidities, previous Hajj/Umrah experience, and uptake of the recommended vaccines.

## Methods

This cross-sectional study is the part of a large quasi-experimental study conducted on Malaysian Hajj and Umrah pilgrims in the year 2018 that are traveling through private hajj companies. The data set in this study was collected from January to November 2018. Various private Hajj companies were approached and invited to participate in the research. Pilgrims from companies who were willing to participate were met at the beginning of the weekly Hajj/Umrah orientation course. Pilgrims were included in the study if they are aged 18 years with or without any chronic medical conditions. The participants were briefed at the start of the session and informed written consent was given to them. A self-administered questionnaire was given to those participants who signed the informed consent form. The survey included pilgrims' demographic information such as age, gender, marital status, occupation, the highest level of education, comorbidities, respiratory illness prior to departing for the Holy Land and history of influenza (flu) vaccine (within the past 1 year) and pneumococcal vaccine (within the past 5 years).

In this study, pilgrims were categorized into “at increased risk” as those in which influenza vaccine and pneumococcal vaccine are strongly recommended i.e., pilgrims aged 50 years and above and/or had pre-existing health conditions such as asthma, diabetics, lung, or kidney diseases (28). Aside these, other pilgrims and those less than the age of 50 years were classified as “non-risk pilgrims.”

### Data Analysis

Data were inserted and cleaned using SPSS version 24.0 (SPSS, Chicago, IL, USA). Descriptive statistics were analyzed using frequencies and percentage for a categorical variable, the mean and standard deviation for continuous variables. Univariable and multivariable analyses were carried out using simple and multiple logistic regression, respectively to examine the relationship between the outcome variables (influenza and pneumococcal vaccines' uptake) and selected socio-demographic factors. Crude odds ratio, adjusted odds ratio (OR) and 95% confidence intervals (CIs) were used as indicators of the strength of association. A *p*-value of 0.05 was used as the level for statistical significance.

### Ethical Consideration

This study got ethical approval from the Human Research Ethics Committee of Universiti Sains Malaysia (ref no: USM/JEPeM/17020146) and Universiti Sultan Zainal Abidin Malaysia human research ethics committees (ref no: UniSZA/UHREC/2019/88) before this study. The data were treated with confidentiality and the results did not identify the respondents personally.

## Results

In all, 1,274 of the 1,500 participants recruited from January to November 2018 returned the questionnaires after completing it (response rate 84.9%). The age of the pilgrims range from 18 to 80 years with mean age with a standard deviation of 42.42 (15.67) and they are predominantly of Malay ethnic group 1,250 (98.7%). The proportion of “at increased risk” pilgrims was 527 (41.4%). Majority of the pilgrims 1,250 (98.7%) were Malay and 833 (65.4) of the respondents were female. In total, 245/1,274 (19.5%) of the respondents reported receiving all the recommended vaccines, while 628 (50.0%) did not receive any of the vaccination. However, 918 (75.0%) did not report any comorbidities. The characteristics of the pilgrims are summarized in Table 1.

**Table 1:** Demographic data of participants and their vaccines uptake.

**Variables**	***n* (%) 1,274**	**Influenza vaccine n (%)**	**Pneumococcal vaccine n (%)**
Age category			
At risk	527 (41.4)	168 (31.9)	184 (34.9)
Not at risk	747 (58.6)	196 (26.2)	140 (18.7)
Gender			
Female	833 (65.4)	211 (25.3)	182 (21.8)
Male	441 (34.6)	153 (34.7)	142 (32.2)
Ethnicity			
Malay	1257 (98.7)	359 (28.7)	321 (25.5)
Indian	5 (0.4)	0(0)	0
Others	12 (0.9)	3(25.0)	3 (25.0)
Marital status			
Single	430 (33.8)	95 (22.1)	64 (14.9)
Married	809 (63.5)	258 (32.0)	248 (30.7)
Divorced/widow	35 (2.7)	11 (31.4)	12 (34.3)
Occupation			
Civil servant	323 (25.4)	67 (20.7)	67 (20.7)
Self employed	105 (8.2)	28 (27.2)	31 (30.1)
Private sector	443 (34.8)	128 (29.2)	97 (22.1)
Pensioner	172 (13.5)	64 (38.1)	70 (41.7)
House wife	128 (10.0)	41 (32.0)	41 (31.3)
Student	103 (8.1)	32 (32.7)	17 (17.3)
Education level			
Bachelor	296 (23.2)	62 (20.9)	55 (18.6)
Diploma	381 (29.9)	124 (32.6)	94 (24.7)
Master	92 (7.2)	23 (25.0)	23 (25.0)
PhD	23 (1.8)	4 (17.4)	1 (4.3)
Secondary	414 (32.5)	137 (33.3)	136 (33.1)
Primary	68 (5.3)	14 (20.6)	15 (22.1)
Previous Hajj experience	450 (35.3)	124 (27.6)	111 (24.7)
Previous Umrah experience	554 (43.5)	174 (24.7)	158 (28.5)
Presence of Comorbidities			
Chronic Lung disease	14 (1.1)	324 (26.7)	6 (42.9)
Neuromuscular disease	56 (4.4)	13 (23.2)	18 (32.1)
Allergic rhinitis	30 (2.4)	11 (36.7)	8 (26.7)
Diabetes	95 (7.5)	37 (38.9)	42 (44.2)
Hypertension	222 (17.4)	77 (34.7)	74 (33.3)
Heart disease	19 (1.5)	8 (42.1)	8 (42.1)
Immune deficiency disorder	10 (0.8)	4 (40.0)	3 (30.0)
Chronic kidney disease	4 (0.3)	2 (50.0)	2 (50.0)
Symptoms of respiratory illness before departure	101 (7.9)	38 (37.6)	27 (26.7)

### Influenza Vaccine Uptake

Overall, 364 (28.6%) of the pilgrims received influenza vaccine. Based on the result from univariable logistic regression analysis, gender, marital status, occupation, educational level, previous Umrah experience and some comorbidities were significant predictors of vaccine uptake. Male pilgrims had 47.9% higher odds of vaccine uptake than females (AOR = 1.47, 95% CI = 1.09–2.00 *p* = 0.011). In addition, marital status, level of educational qualification as well as occupation and were also significant predictors for vaccine uptake. Pilgrims that are single have 61% fewer chances of vaccine uptake. However, self-employed pilgrims and diploma holder had 48 and 84% higher chances of getting vaccinated than civil servants (AOR = 1.48, 95% CI = 0.82–2.67) and bachelor degree (AOR = 1.84, 95% CI = 1.25–2.70) as shown in Table 2.

**Table 2:** Significant factors associated with vaccines uptake among Malaysian Hajj/Umrah pilgrims.

**Predictors**	**Crude OR^**a**^**	**Adjusted OR^**b**^**	**95% CI**	***p*-value**
Influenza vaccine				
Gender				
Female	1	1		
Male	1.57	1.486	1.11–1.99	0.008
Marital Status				
Married	1	1		
Single	0.60	0.37	0.25–0.55	<0.001
Divorced/widowed	0.98	1.08	0.46–2.56	0.85
Occupation				
Civil servant	1	1		
Self employed	1.43	1.35	0.76–2.40	0.309
Private sector	1.57	2.39	1.59–3.58	<0.001
Pensioner	2.35	1.95	1.25–3.03	0.003
House wife	1.80	1.42	0.80–2.56	0.230
Student	1.85	3.24	1.62–6.49	0.001
Educational level				
Bachelor	1	1		
Diploma	1.83	1.90	1.30–2.79	0.001
Master	1.26	1.24	0.69–2.22	0.464
PhD	0.80	0.69	0.21–2.20	0.528
Secondary	1.89	1.25	0.84–1.87	0.264
Primary	0.98	0.51	0.23–1.16	0.107
Previous Umrah experience				
Yes	1	1		
No	1.42	1.513	1.16–1.98	0.003
Symptoms of respiratory tract infections before departure				
No	1	1		
Yes	0.58	0.472	0.30–0.75	0.001
Pneumococcal vaccine				
Gender				
Female	1	1		
Male	1.72	1.67	1.24–2.25	0.001
Marital status				
Married	1	1		
Single	0.39	0.407	0.27–0.62	<0.001
Divorced/widow	1.15	0.906	0.40–2.05	0.813
Occupation				
Civil servant	1	1		
Self employed	1.65	1.70	0.98–2.93	0.058
Private sector	1.09	1.86	1.24–2.80	0.003
Pensioner	2.75	2.29	1.48–3.54	<0.001
House wife	1.77	1.83	1.09–3.07	0.022
Student	0.82	1.92	0.93–3.96	0.080
Previous Umrah experience				
No	1	1		
Yes	1.43	1.48	1.12–1.95	0.006
Diabetes				
No	1	1		
Yes	0.36	0.50	0.31–0.80	0.004

a *Simple logistic regression*.

b *Multiple logistic regression*.

### Pneumococcal Vaccine Uptake

The Overall, only 25.4% (324) of the respondents reported receiving the pneumococcal vaccine. The coverage varied within the age category with those “at risk” having 35.6% (143) and those “not at risk” having 19.4% (143). The uptake of the pneumococcal vaccine among marital status were 64 (14.9%), 248 (30.7), and 12 (34.3) for single, married and divorced/widowed pilgrims, respectively. Univariable logistic regression analysis of the predictors for vaccine uptake showed gender, marital status, occupation, and previous Umrah experience as the positive predictors. Based on multivariate analysis showed that being male (AOR = 2.15, 95% CI: 1.49–3.09, *P* < 0.01), self-employed (AOR = 1.58, 95% CI: 0.93–2.71, *P* = 0.004), a pensioner (AOR = 2.33, 95% CI: 1.53–3.56, *P* = 0.004), and having a previous Umrah experience (AOR = 1.58, 95% CI: 1.20–2.07, *P* < 0.001) increases the odds of pneumococcal vaccine uptake. However, being single (AOR = 0.37, 95% CI: 0.25–0.56, *P* < 0.001) and divorced/widowed (AOR = 0.91, 95% CI: 0.41–2.05) reduces the odds of being vaccinated against pneumococcal disease (Table 2).

## Discussion

The primary objective of this research was to assess the uptake of influenza and pneumococcal vaccine and identify the factors associated with the uptake of these vaccines. This is due to the enormous role Hajj and Umrah play in global public health due to the risk of importing and spreading new infections to home country on return. The findings from this study revealed that the uptake of these vaccines was significantly higher among pilgrims with higher education levels. This study also showed that 41.4% of the participants are considered to be at risk of respiratory tract infection and the vaccine uptake among this group are 31.9 and 34.9% for influenza and pneumococcal vaccine, respectively. This findings are similar to the finding of a study conducted among pilgrims sample on arrival at King Abdulaziz International Airport in Jeddah for the 2009 Hajj (29). However, the uptake of these vaccines among this group is lower than that in a previous study conducted among pilgrims from Saudi (67%) (30). Despite the regulations by the Saudi health authority that necessitate influenza and pneumococcal vaccine compulsory before departure to Hajj, 100% coverage regarding the uptake of the vaccine is still lacking among most pilgrims from various countries (30–32). Our present study also showed a similar low level of recommended vaccines uptake among Malaysian Hajj and Umrah pilgrims. The low uptake could be explained based on the fact that pandemic influenza vaccine is not given free by the Health ministry, most are available at private clinics only a short time before the departure of pilgrims from their home countries and costly as reported in other similar studies (33). Also, the suboptimal acceptance of these vaccines as reported in this study may have added to such lower vaccination coverage among pilgrims (34).

Results from this study showed that the most common medical condition among the pilgrims was hypertension (17.9%) followed by diabetes (7.7%). Similarly, few pilgrims reported symptoms of respiratory tract infections (%) before departure. Despite these numbers of pilgrims with chronic medical conditions, a low proportion of them (19.5%) reported having all the recommended vaccines. Pilgrims with comorbidities are considered as at increased risk of respiratory tract infections (25).

A study done in Malaysia in 2013 Hajj season showed vaccination coverages for the influenza virus and pneumococcal disease at 65.2 and 59.4%, respectively (9). These are quite high when compared with the present study, which reported influenza and pneumococcal vaccine uptake at 28.6 and 25.4%. However, the low influenza vaccine uptake in our study are comparable to studies conducted among French pilgrims (31.8%), Turkish pilgrims (7.1%), and pilgrims from Saudi Arabia, Qatar, and Australia (31.0%) (35–37). Similarly, low pneumococcal vaccine update as reported in this study was published in previous study among Australian pilgrims (32). This variation could be due to the increased awareness and enlightenment following 2009/2010 influenza pandemic and the emergence of Middle East respiratory syndrome coronavirus (MERS-CoV) in Saudi Arabia in 2012 (38). Pneumococcal vaccine figures posed a severe challenge in the control and prevention of pneumonia considering the high rate of hospital admission and mortality it resulted to during Hajj (39, 40).

Influenza vaccine uptake among females (25.3%) is higher when compared to males (34.7%). This result is contrary to the finding of a study conducted among Australian Hajj pilgrims of 73 and 59% for females and males, respectively (35). Pilgrims with comorbidities are at increased risk of severe diseases especially the vaccine-preventable infections (25). However, pilgrims with such chronic medical conditions are highly recommended to receive the recommended vaccines by health authorities (41). From this study, 18.2% of the pilgrims reported having at least one medical condition and above. Similarly, 41.4% of the whole pilgrims surveyed are also “at risk” based on age category and therefore highly recommended for all the three vaccines before. The current study showed that the pilgrims “at risk” had lower vaccination uptake than the “not at risk” pilgrims at 41.4 and 58.6%, respectively. Majority of the pilgrims “at risk” are more concerned toward pneumococcal vaccine (34.9) than influenza vaccine (31.9%). Alqahtani et al. (3) reported similar findings among Australian Hajj pilgrims. Previous Umrah experience was found to be a significant predictor of all the vaccines uptake (*p* < 0.005).

This study has some limitations which demand some elaboration. Result of our study could not be broadly generalized to the whole of Malaysia as it was conducted in Kelantan state of Malaysia. The data from this study might be liable to recall bias since the period of vaccination for influenza vaccine could be up to 2 years and is based on self-reported vaccination history.

## Conclusion

In conclusion, this study showed that the recommended vaccine's uptake among Malaysian Hajj and Umrah pilgrims in 2018 is still very low based on the World Health Organization (WHO) goal of 75% coverage or higher among the “at increased risk” of infection pilgrims. Education of all pilgrims before departure is imperative to enlighten and create awareness toward vaccine uptake. Additional studies are required to explore the barriers as well as motivators to bridge the knowledge gap about the mandatory and recommended vaccine's uptake.

## Data Availability Statement

The datasets generated for this study are available on request to the corresponding author.

## Ethics Statement

The studies involving human participants were reviewed and approved by Human Research Ethics Committee of Universiti Sains Malaysia, Universiti Sains Malaysia Health Campus, Kelantan Malaysia (ref no: USM/JEPeM/17020146), Universiti Sultan Zainal Abidin (UniSZA) Human Research Ethics Committee (UHREC) (UniSZA/UHREC/2019/88), Malaysia. The patients/participants provided their written informed consent to participate in this study.

## Author Contributions

MG and ZD conducted the survey and drafted the initial manuscript. NN, HH, NW-A, and WA designed and supervised the study. AB helped in statistical analysis, interpretation, and manuscript revision. All authors read and approved the final manuscript.

### Conflict of Interest

The authors declare that the research was conducted in the absence of any commercial or financial relationships that could be construed as a potential conflict of interest.

## References

[1] Memish ZA Al-Tawfiq JA Al-Rabeeah AA. Hajj: preparations underway. Lancet Glob Health. (2013) 1:e331. 10.1016/S2214-109X(13)70079-2 25104593 PMC7129895

[2] Aziz MM El-Megeed HSA Ellatif MAMA. Pre-travel health seeking practices of Umrah pilgrims departing from Assiut International Airport, Egypt. Travel Med Infect Dis. (2018) 23:72–6. 10.1016/j.tmaid.2018.04.012 29689385

[3] Alqahtani AS Rashid H Heywood AE. Vaccinations against respiratory tract infections at Hajj. Clin Microbiol Infect. (2015) 21:115–27. 10.1016/j.cmi.2014.11.026 25682277

[4] Khan ID Khan SA Asima B Hussaini SB Zakiuddin M Faisal F. Morbidity and mortality amongst Indian Hajj pilgrims: a 3-year experience of Indian Hajj medical mission in mass-gathering medicine. J Infect Public Health. (2018) 11:165–70. 10.1016/j.jiph.2017.06.004 28668659 PMC7102688

[5] Al-Tawfiq JA Zumla A Memish ZA. Respiratory tract infections during the annual Hajj: potential risks and mitigation strategies. Curr Opin Pulm Med. (2013) 19:192–7. 10.1097/MCP.0b013e32835f1ae8 23429098

[6] Abubakar I Gautret P Brunette GW Blumberg L Johnson D Poumerol G. Global perspectives for prevention of infectious diseases associated with mass gatherings. Lancet Infect Dis. (2012) 12:66–74. 10.1016/S1473-3099(11)70246-8 22192131

[7] Khan K Sears J Hu VW Brownstein JS Hay S Kossowsky D. Potential for the international spread of middle East respiratory syndrome in association with mass gatherings in Saudi Arabia. PLoS Curr. (2013) 5. 10.1371/currents.outbreaks.a7b70897ac2fa4f79b59f90d24c860b8 23884087 PMC3714242

[8] Deris ZZ Hasan H Sulaiman SA Wahab MSA Naing NN Othman NH. The prevalence of acute respiratory symptoms and role of protective measures among Malaysian Hajj pilgrims. J Travel Med. (2010) 17:82–8. 10.1111/j.1708-8305.2009.00384.x 20412173

[9] Hashim S Ayub ZN Mohamed Z Hasan H Harun A Ismail N. The prevalence and preventive measures of the respiratory illness among Malaysian pilgrims in 2013 Hajj season. J Travel Med. (2016) 23:tav019. 10.1093/jtm/tav019 26858268 PMC7107508

[10] Yezli S. The threat of meningococcal disease during the Hajj and Umrah mass gatherings: a comprehensive review. Travel Med Infect Dis. (2018) 24:51–8. 10.1016/j.tmaid.2018.05.003 29751133

[11] El Ghany MA Sharaf H Hill-Cawthorne GA Hajj vaccinations—facts, challenges, and hope. Int J Infect Dis. (2016) 47:29–37. 10.1016/j.ijid.2016.05.024 27260241

[12] Kandeel A Deming M Kereem EA El-Refay S Afifi S Abukela M. Pandemic (H1N1) 2009 and Hajj Pilgrims who received predeparture vaccination, Egypt. Emerg Infect Dis. (2011) 17:1266–8. 10.3201/eid1707.101484 21762583 PMC3381419

[13] Azeem MI Tashani M Badahdah AM Heron L Pedersen K Jeoffreys N. Surveillance of Australian Hajj pilgrims for carriage of potentially pathogenic bacteria: data from two pilot studies. World J Clin Cases. (2017) 5:102–11. 10.12998/wjcc.v5.i3.102 28352634 PMC5352958

[14] Pleban FT Mass gatherings and hazard control: agenda for education and implementation. Handb Healthc Arab World. (2019) 1–22. 10.1007/978-3-319-74365-3_51-1

[15] Memish ZA Zumla A Alhakeem RF Assiri A Turkestani A Al Harby KD. Hajj: infectious disease surveillance and control. Lancet. (2014) 383:2073–82. 10.1016/S0140-6736(14)60381-0 24857703 PMC7137990

[16] Malaysian Clinical Practical Guidelines AoMoM Clinical Practice Guidelines, Adult Vaccination. Kuala Lumpur: Academy of Medicine of Malaysia (2010).

[17] Hajj T Immunization Information: Tabung Hajj. (2019). Available online at: https://www.tabunghaji.gov.my/en/immunization-information (accessed July 27, 2019).

[18] Tabung Hajj Hajj Quota. (2019). Available online at: https://www.tabunghaji.gov.my/en/hajj/general-info/hajj-quota (accessed July 27, 2019).

[19] Zafer N Dulong C Rahman A Tashani M Alfelali M Alqahtani AS. Acute respiratory tract infection symptoms and the uptake of dual influenza and pneumococcal vaccines among Hajj pilgrims. Int Marit Health. (2018) 69:278–84. 10.5603/IMH.2018.0044 30589068

[20] Alborzi A Aelami MH Ziyaeyan M Jamalidoust M Moeini M Pourabbas B. Viral etiology of acute respiratory infections among Iranian Hajj pilgrims, 2006. J Travel Med. (2009) 16:239–42. 10.1111/j.1708-8305.2009.00301.x 19674262

[21] Emamian MH Hassani AM Fateh M. Respiratory tract infections and its preventive measures among Hajj pilgrims, 2010: a nested case control study. Int J Prev Med. (2013) 4:1030–5. 24130944 PMC3793484

[22] Meysamie A Ardakani HZ Razavi SM Doroodi T. Comparison of mortality and morbidity rates among Iranian pilgrims in Hajj 2004 and 2005. Saudi Med J. (2006) 27:1049–53. 16830029

[23] Shafi S Rashid H Ali K El Bashir H Haworth E Memish ZA. Influenza vaccine uptake among British Muslims attending Hajj, 2005 and 2006. BMJ. (2006) 333:1220. 10.1136/bmj.39051.734329.3A 17158396 PMC1693606

[24] Moattari A Emami A Moghadami M Honarvar B. Influenza viral infections among the Iranian Hajj pilgrims returning to Shiraz, Fars province, Iran. Influenza Other Respir Viruses. (2012) 6:e77–9. 10.1111/j.1750-2659.2012.00380.x 22606996 PMC4941701

[25] Alfelali M Barasheed O Badahdah AM Bokhary H Azeem MI Habeebullah T. Influenza vaccination among Saudi Hajj pilgrims: revealing the uptake and vaccination barriers. Vaccine. (2018) 36:2112–8. 10.1016/j.vaccine.2018.03.007 29555221 PMC7115686

[26] Haridi H Salman K Basaif E Al-Skaibi D. Influenza vaccine uptake, determinants, motivators, and barriers of the vaccine receipt among healthcare workers in a tertiary care hospital in Saudi Arabia. J Hosp Infect. (2017) 96:268–75. 10.1016/j.jhin.2017.02.005 28283372

[27] Taib WRW Yusoff NAM Hussin TMAR Ahmad A Issues in vaccine hesitancy in Malaysia: a countering approach. J Biomed Clin Sci. (2017) 2:42–6.

[28] Malaysia AoMo Clinical Practice Guidelines, Adult Vaccination. Kuala Lumpur: Academy of Medicine of Malaysia (2019).

[29] Memish ZA Assiri AM Hussain R Alomar I Stephens G. Detection of respiratory viruses among pilgrims in Saudi Arabia during the time of a declared influenza A (H1N1) pandemic. J Travel Med. (2011) 19:15–21. 10.1111/j.1708-8305.2011.00575.x 22221807 PMC7537650

[30] Alqahtani AS Althimiri NA BinDhim NF. Saudi Hajj pilgrims' preparation and uptake of health preventive measures during Hajj 2017. J Infect Public Health. (2019). 10.1016/j.jiph.2019.04.007. [Epub ahead of print]. 31023600

[31] Gautret P Bauge M Simon F Benkouiten S Parola P Brouqui P. Pneumococcal vaccination and Hajj. Int J Infect Dis. (2011) 15:e730. 10.1016/j.ijid.2011.07.001 21840741

[32] Alqahtani AS Wiley KE Tashani M Willaby HW Heywood AE BinDhim NF. Exploring barriers to and facilitators of preventive measures against infectious diseases among Australian Hajj pilgrims: cross-sectional studies before and after Hajj. Int J Infect Dis. (2016) 47:53–9. 10.1016/j.ijid.2016.02.005 26875699 PMC7110465

[33] Khan K Memish ZA Chabbra A Liauw J Hu W Janes DA. Global public health implications of a mass gathering in Mecca, Saudi Arabia during the midst of an influenza pandemic. J Travel Med. (2010) 17:75–81. 10.1111/j.1708-8305.2010.00397.x 20412172

[34] Ahmed GY Balkhy HH Bafaqeer S Al-Jasir B Althaqafi A. Acceptance and adverse effects of H1N1 vaccinations among a cohort of national guard health care workers during the 2009 Hajj season. BMC Res Notes. (2011) 4:61. 10.1186/1756-0500-4-61 21396123 PMC3063226

[35] Barasheed O Rashid H Alfelali M Tashani M Azeem M Bokhary H. Viral respiratory infections among Hajj pilgrims in 2013. Virol Sin. (2014) 29:364–71. 10.1007/s12250-014-3507-x 25413828 PMC7090649

[36] Benkouiten S Charrel R Belhouchat K Drali T Nougairede A Salez N. Respiratory viruses and bacteria among pilgrims during the 2013 Hajj. Emerg Infect Dis. (2014) 20:1821–7. 10.3201/eid2011.140600 25341199 PMC4214309

[37] Gautret P Benkouiten S Griffiths K Sridhar S. The inevitable Hajj cough: surveillance data in French pilgrims, 2012–2014. Travel Med Infect Dis. (2015) 13:485–9. 10.1016/j.tmaid.2015.09.008 26464001 PMC7110682

[38] Al-Tawfiq JA Zumla A Memish ZA. Travel implications of emerging coronaviruses: SARS and MERS-CoV. Travel Med Infect Dis. (2014) 12:422–8. 10.1016/j.tmaid.2014.06.007 25047726 PMC7110592

[39] Ridda I King C Rashid H. Pneumococcal infections at Hajj: current knowledge gaps. Infect Disord Drug Targets. (2014) 14:177–84. 10.2174/1871526514666141014150323 25313100

[40] Al-Tawfiq JA Memish ZA. Prevention of pneumococcal infections during mass gathering. Hum Vaccin Immunother. (2016) 12:326–30. 10.1080/21645515.2015.1058456 26176306 PMC5049738

[41] Badahdah AM Alfelali M Alqahtani AS Alsharif S Barasheed O Rashid H. Mandatory meningococcal vaccine, and other recommended immunisations: uptake, barriers, and facilitators among health care workers and trainees at Hajj. World J Clin Cases. (2018) 6:1128–35. 10.12998/wjcc.v6.i16.1128 30613671 PMC6306626

